# Ultrabroadband direct detection of nonclassical photon statistics at telecom wavelength

**DOI:** 10.1038/srep04535

**Published:** 2014-04-03

**Authors:** Kentaro Wakui, Yujiro Eto, Hugo Benichi, Shuro Izumi, Tetsufumi Yanagida, Kazuhiro Ema, Takayuki Numata, Daiji Fukuda, Masahiro Takeoka, Masahide Sasaki

**Affiliations:** 1 National Institute of Information and Communications Technology (NICT), 4-2-1 Nukui-kitamachi, Koganei, Tokyo 184-8795, Japan; 2 Department of Physics, Gakushuin University, Mejiro 1-5-1, Toshima-ku, Tokyo, 171-8588, Japan; 3 Department of Engineering and Applied Sciences, Sophia University, 7-1 Kioi-cho, Chiyoda-ku, Tokyo 102-8554, Japan; 4 National Institute of Advanced Industrial Science and Technology (AIST), 1-1-1 Umezono, Tsukuba, Ibaraki 305-8568, Japan; 5 Quantum Information Processing Group, Raytheon BBN Technologies, 10 Moulton Street, Cambridge, MA 02140, USA

## Abstract

Broadband light sources play essential roles in diverse fields, such as high-capacity optical communications, optical coherence tomography, optical spectroscopy, and spectrograph calibration. Although a nonclassical state from spontaneous parametric down-conversion may serve as a quantum counterpart, its detection and characterization have been a challenging task. Here we demonstrate the direct detection of photon numbers of an ultrabroadband (110 nm FWHM) squeezed state in the telecom band centred at 1535 nm wavelength, using a superconducting transition-edge sensor. The observed photon-number distributions violate Klyshko's criterion for the nonclassicality. From the observed photon-number distribution, we evaluate the second- and third-order correlation functions, and characterize a multimode structure, which implies that several tens of orthonormal modes of squeezing exist in the single optical pulse. Our results and techniques open up a new possibility to generate and characterize frequency-multiplexed nonclassical light sources for quantum info-communications technology.

Discriminating the number of incoming photons shot-by-shot plays an indispensable role in quantum information processing (QIP)^1^,^2^,^3^, and quantum communication^4^,^5^,^6^. Photon number resolving detection (PNRD) is a highly nonlinear process, and hence can be used, combined with nonclassical light sources, for quantum state preparation^7^,^8^ and to implement quantum photonic gates^1^,^2^,^3^. Devices for PNRD, such as avalanche photodiode (APD) for the time-multiplexing scheme^9^ and superconducting transition-edge sensor (TES)^10^,^11^,^12^,^13^, have broadband sensitivity, covering most of the spectra of spontaneous parametric down-conversion (SPDC), which is an established source of nonclassical light^14^,^15^. SPDC sources inherently generate broadband multimode nonclassical states^16^,^17^,^18^. If appropriate mode engineering can be made in those multimode states, the implementation of QIP^19^,^20^ can potentially be more scalable.

Multimode nonclassical light sources and their characterization have been studied extensively these years. In the frequency domain, quantum continuous variable (CV) correlations in the frequency comb were generated using a single optical parametric oscillator (OPO) and characterized for many sideband modes by homodyne detectors with designated local oscillator (LO) modes^21^,^22^. In the time domain, CV entanglement over massive temporal modes, was created by using two OPOs and a delayed interferometer^23^. In the spatial domain, CV entanglement over multiple transverse modes was generated from an OPO and characterized by homodyning^24^,^25^.

Besides these studies with CVs, discrete variable (DV) nature, i.e., photon-number statistics, of multimode quantum states have been investigated and characterized in this decade, by using PNRDs. The direct observation of nonclassical photon-number oscillation from a type-I SPDC source was demonstrated using a visible light photon counter (VLPC)^26^,^27^. The higher order photon-number correlations between the signal and idler beams of a twin beam from a type-II SPDC source were observed, and the influence of multimode structure to photon-number statistics was analysed, using the APD-based time-multiplexing PNRD^28^,^29^. Extending this idea, reconstruction of the mode weight structure from the photon-number correlations was also demonstrated for the states containing three modes^30^. All of these works with CVs and DVs have been performed at the visible or near-infrared wavelengths.

In this paper, we demonstrate the direct detection of nonclassical photon statistics of ultrabroadband squeezed states at the telecom wavelength. The squeezed states are generated from a SPDC source and their broadband multimode photon-number statistics is measured by a highly efficient PNRD based on a titanium TES^11^,^12^. The centre wavelength of the generated state is 1535 nm and the measured bandwidth is as broad as 110 nm (13.4 THz), covering the S-, C-, and L-bands which are major bands for optical fibre communication. We characterize the nonclassical property by the following two observations: first, we directly measure the photon-number distribution of a whole multimode state and confirm its nonclassicality by Klyshko's criterion^26^,^31^. Second, we apply the method proposed by Christ *et al.*^18^ and reconstruct the mode weight distribution, i.e. the degrees of squeezing in each decomposed mode from the higher order photon-number correlations, which implies that several tens of independent squeezers are contained in our source. The results and techniques pave a new simple way of characterizing ultrabroadband nonclassical states and we anticipate it will accelerate further research and development on quantum information technologies for the telecom fibre infrastructures.

## Results

### Broadband squeezed light source

Our light source is a single-beam squeezer in a collinear configuration where the pump, signal, and idler beams are in the same spatiotemporal and polarization mode. The generation process is mathematically described by the following unitary transformation^18^,^32^

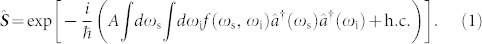
Here *A* denotes the overall efficiency of the squeezing, *f* (*ω*_s_, *ω*_i_) is the joint-spectral distribution (JSD) in terms of the signal and idler angular frequencies, *ω*_s_ and *ω*_i_, and
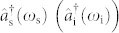
 represents the creation operator of the signal (idler) field in the continuous spectrum of *ω*_s_ (*ω*_i_). These continuous modes are, however, not necessarily a convenient representation to deal with the multimode characteristics, because they cannot be decoupled from each other for most of practical JSDs. For a JSD which distributes in an effectively finite range, one can find a more convenient discrete set of decoupled modes^32^,^33^,^34^,^35^,^36^.

In fact, when the JSD is engineered to be symmetric, which is the case here, it can then be decomposed into the following form 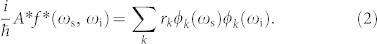
in terms of a complete orthonormal set {*ϕ_k_*(*ω*)}^18^. Note that each *ϕ_k_*(*ω*) represents the shape of the frequency distribution for signal and idler. Here *r_k_* is a modal amplitude corresponding to a squeezing parameter for mode *k*. Introducing the field operators for the broadband basis modes^36^, 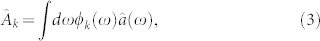
the above transformation can be expressed as 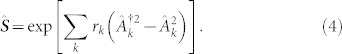
The generated state is a multimode squeezed vacuum state as 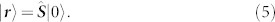
Thus this state only includes even number of photons. In practice, however, inevitable losses in the generation, propagation, and detection processes cause the vacuum invasion, making the pure state |***r***〉 a mixed one, whose statistics has odd number components as well. If losses are below a certain level, one can still observe even-rich photon-number statistics. Direct observation of this even-odd number oscillation and multimode analysis on the distribution of *r_k_* are our tasks here.

### Experimental setup

**Figure 1:**
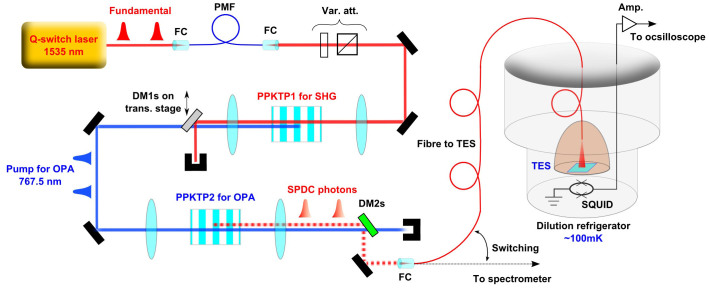
Experimental setup. Schematic diagram of the experimental setup. The fundamental light is guided into a polarization-maintaining fibre (PMF, used as a spatial-mode filter) via a fibre-coupler (FC), passes through variable attenuator (Var. att.) consisting of a half wave plate and a polarizing beam splitter, and is then injected into a PPKTP crystal for SHG. The SHG output pumps another PPKTP crystal for squeezing. DM1s (DM2s) are dichroic mirrors acting as a high reflector for the fundamental (pump) light and as a high transmitter for the pump (fundamental) light. SPDC output is coupled into an optical fibre and guided to a TES or a spectrometer.

Figure 1 shows the experimental setup. The fundamental light source is a single-longitudinal-mode, passively Q-switched, diode-pumped solid-state laser (Cobolt, Tango), operating at 1535 nm with a pulse width of 4 ns and a repetition rate of 3.1 kHz. This is used as the fundamental light, and is injected into a 10 mm long, periodically poled KTiOPO_4_ (PPKTP) crystal for second harmonic generation (SHG) at 767.5 nm. This second harmonic light is then used as a pump for squeezing. An unconverted fundamental light after SHG is used as a guiding beam for fibre-coupling to the TES, but effectively eliminated by dichroic mirrors when photon counting experiment is implemented. The pump is guided into the second type-0 PPKTP crystal employed as the squeezer. The PPKTP crystals for SHG and the squeezer are mounted on copper stages and temperature-stabilized. The multimode squeezed vacuum state is fibre-coupled and guided to the TES or to a spectrometer. Our TES is based on a titanium superconductor whose size is 10 × 10 *μ*m^2^ as shown in the reference^11^,^12^, and is set inside a dilution refrigerator (TS-3H100-PT, Taiyo Nippon Sanso). Output waveform from the TES is amplified by a SQUID device and room-temperature electronics (Magnicon). Each waveform was sampled using a digital oscilloscope (DPO7104, Tektronix), and sent to a host computer for noise filtering and data analysis.

**Figure 2:**
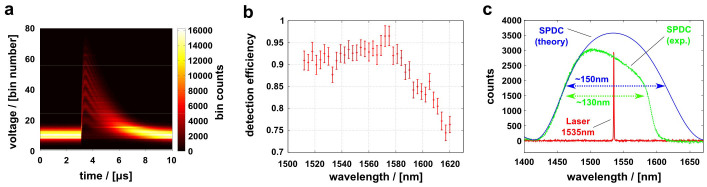
TES specifications and SPDC spectrum. (a) Typical waveforms from the TES. Total 1 × 10^7^ waveforms are overlaid. The vertical direction is divided into bins and voltage counts are summed with respect to each bin. Each waveform contains 1000 points (horizontal direction). (b) Wavelength dependence of TES's detection efficiency from 1512 nm to 1620 nm. (c) Spectrum of the SPDC. The red line shows spectrum of the fundamental laser source. The green dotted and blue lines are measured and theoretical spectra of SPDC, respectively.

Figure 2a shows raw waveforms from the TES. Total 1 × 10^7^ waveforms are stored and overlaid here. The gating time for each detection event was 10 microseconds. Within this gating period, an average photon number of TES's dark counts was measured to be 5 × 10^−4^ per pulse, which corresponds to 50 counts per second. The wavelength dependence of our TES's DE is shown in Fig. 2b (also see Methods). It is almost flat from 1512 nm to 1580 nm, and then gradually drops from 1580 nm to 1620 nm. The absolute DE is = 90.9 ± 2.4% at 1535 nm. Unfortunately, we were not able to measure DE at wavelengths shorter than 1510 nm nor longer than 1620 nm, because of a limited wavelength range of the probe light.

The red line in Fig. 2c is a spectrum of the fundamental light, whose linewidth is ~0.05 nm. The green dotted line is a spectrum of the SPDC measured with a spectrometer (Acton) for a 30 sec accumulation time. Measured full-width at half maximum (FWHM) is roughly 130 nm. The blue line is a theoretically derived spectrum using the present experimental parameters (see Methods), which is symmetric about the fundamental light spectrum. The asymmetry seen in the measured spectrum (green dotted line) is an artifact caused by the DE fall-off of an InGaAs photo detector in the spectrometer. Actually the spectrometer DE gradually drops when the wavelength exceeds 1500 nm. Then the DE starts steeply falling at around 1580 nm and reaches almost 0% at around 1650 nm. The blue theoretical spectrum captures the actual SPDC spectrum whose FWHM extends over almost 150 nm in the telecom window, covering the S-, C-, and L-bands.

### Nonclassical photon statistics of multimode squeezed states

**Figure 3:**
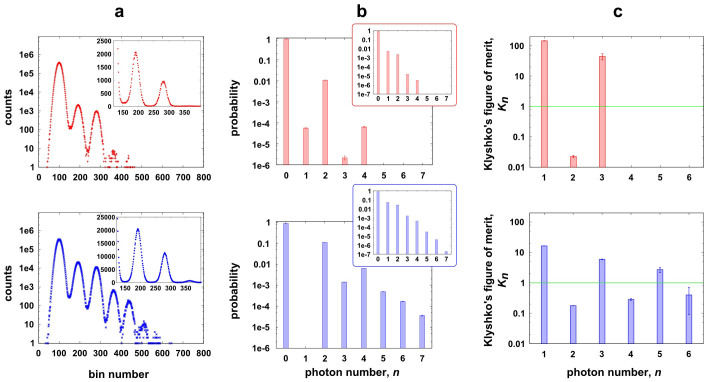
Photon statistics of multimode squeezed states. (a) Pulse height distributions of TES output waveforms for two different pump conditions: ~1 pJ for the upper panel and ~10 pJ for the lower panel, respectively. The main graphs are in log scale, while the insets are in linear scale. (b) Reconstructed photon-number distributions with the loss and dark conts compensated. The insets are raw photon-probability distribution in log scale for the vertical axes. (c) Klyshko's figure of merit in log scale. All the error bars in (b) and (c) were statistical errors calculated by linear error-propagation method. We assume that photon-counting events obey Poissonian statistics.

Figure 3 shows the experimental results for the multimode squeezed states. Through 3a, b, and c, the histograms in red and blue correspond to a pump pulse energy of ~1 pJ and ~10 pJ, respectively. Figure 3a shows the pulse height distribution of our Ti-TES output waveforms. A voltage range of −20 to 60 mV is divided into 800 bins. The total number of events was 1 × 10^7^ for each data set. The vertical axes in the main graphs are numbers of counts shown in log scale, while those in the insets are in linear scale. Every peak in each distribution was clearly separated, and was fitted with a Gaussian function in order to determine thresholds for calculating photon-number probabilities. The FWHM of the each Gaussian peak corresponds to an energy resolution and was calculated to be about 0.2 eV, which is smaller enough compared with a photon energy (~0.8 eV) of the monochromatic fundamental light at 1535 nm.

Figure 3b insets show the photon-number distributions obtained from the pulse height distributions without any correction of losses. In each condition, the results clearly show super-Poissonian distribution, whose feature could be confirmed by the Fano factors, which are greater than the unity, yielding 1.462 ± 0.002 (red) and 1.494 ± 0.001 (blue), as well as by *g*^(2)^ values, 42.746 ± 0.195 (red) and 4.978 ± 0.008 (blue). Then, the overall DE including the fibre-coupling efficiency can be evaluated by comparing the single- and two-photon probabilities, according to the formula, 
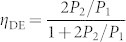
27. The overall DE was thus estimated to be *η*_DE_ = 48.4 ± 0.2%. Note the dark counts slightly changed *P*_1_, and we therefore subtracted its contribution from the raw statistics for calculating DE.

The main graphs in Fig. 3b are reconstructed photon-number statistics using the iterative maximum-likelihood (ML) estimation algorithm^37^. Through the ML estimation process, we used the positive operator-valued measure (POVM) of our TES described as follows^38^, 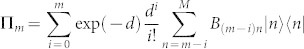, where 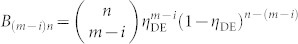. The POVM elements are normalized in order to fulfill Σ*_m_* Π*_m_* = **I**. TES is thus modelled as a linear photon counter which detects *m* photons from *n* input ones with the finite detection efficiency *η*_DE_, and with the dark counts per pulse *d*. *M* is a cutoff photon-number chosen to be large enough in order to prevent estimation errors by truncating higher photon-numbers. We chose *M* = 10 here. The reconstructed statistics were fully converged after 1 × 10^6^ iterations of the ML estimation algorithm. Note, in our case, the linear inversion of the binomial distribution *B*_(*m*−*i*)*k*_ resulted in unphysical, negative photon probabilities because of alternating signs in 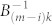 components^39^. The reconstructed two-photon probability *P*_2_ in red (upper panel) is about 1%. On the other hand, the reconstructed two- and four-photon probabilities for the stronger pumping condition (lower panel in blue) resulted *P*_2_ ~ 10% and *P*_4_ ~ 0.6%, respectively. Thus the reconstructed probabilities exhibit even-rich photon-number statistics due to the pairwise photon generation of squeezing.

We also evaluated the nonclassicality using Klyshko's criterion^26^,^31^. Klyshko defined the figure of merit 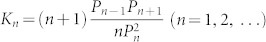 and showed that for any {*P_n_*} originating from classical states, such as coherent state or thermal state, *K_n_* ≥ 1 should be satisfied for all *n*^31^. In other words, if this inequality is violated for any of *K_n_*, the distribution {*P_n_*} is necessarily from a nonclassical state. In Fig. 3c, *K_n_* were calculated from the raw photon distribution (Fig. 3b insets), and the classical limit (green horizontal line) is clearly violated (*K_n_* < 1) for even-photon numbers (*n* = 2, 4, and 6), while all the odd photon-numbers do not violate the limit. Ideally, squeezed source consists of even-photon numbers and therefore all the *K_n_* for odd numbers must go infinite. There were in practice some contributions of odd-photon numbers caused by the finite DE, resulting in the finite *K_n_* for odd photon-numbers.

### Mode analysis of multimode squeezed states

**Figure 4:**
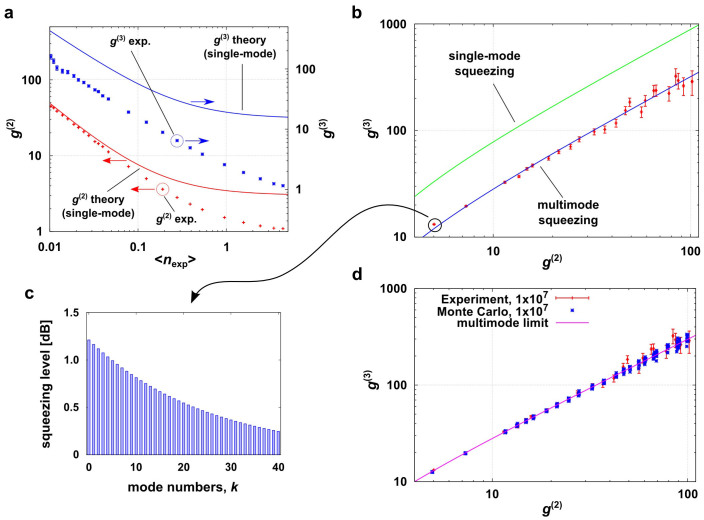
Mode analysis of multimode squeezed states. (a) Experimental *g*^(2)^ and *g*^(3)^. They show clear deviations from the single-mode cases (solid lines). (b) Experimental *g*^(3)^ are plotted as a function of experimental *g*^(2)^ (red crosses). The blue solid line is a result of fitting the data. From a slope of the fitted curve, we obtained *μ*_exp_, that determines a shape of the squeezer distribution. The green solid line is the single-mode case, *g*^(3)^ = 9*g*^(2)^ − 12, for reference. (c) is a reconstructed squeezing-level distribution using *μ*_exp_ and *B*_exp_ for a pump energy of ~10 pJ. (d) Result of Monte Carlo simulations. The blue crosses are *g*^(2)^ and *g*^(3)^ obtained by the simulations. The red crosses are the experimental data replotted for comparison. The magenta line is multimode limit shown as a reference.

Using the above PNRD data, we evaluate experimental *g*^(2)^ and *g*^(3)^, and analyse multimode structures of our squeezer by applying the method in the ref. 18. Figure 4a shows experimental *g*^(2)^ and *g*^(3)^ calculated from the photon-number statistics using *m*th factorial moment *n*^(*m*)^ given by 
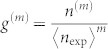
, (*m* = 2 or 3) where 
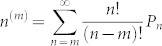
 and 
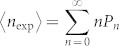
40. The red and blue solid lines are theoretical plots for the single-mode squeezed state given by 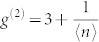, 
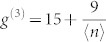
. Because *g*^(*m*)^ is a loss-tolerant measure, we use 〈*n*〉 = 〈*n*_exp_〉/*η*_DE_ in order to compare the experimental *g*^(2)^ and *g*^(3)^ to the theory. As expected from the fact that our squeezed states are highly multimode, our experimental *g*^(2)^ and *g*^(3)^ show clear deviations from the single-mode theory. This can be contrasted to an experimental result on single-mode squeezed states from a PPKTP, which showed that the experimental *g*^(2)^ agreed well with the single-mode theory^41^.

It is generally a nontrivial task to carry out the Schmidt decomposition analytically. For certain classes of states, however, it can be made. A typical class is a two-dimensional (2D) JSD *f*(*ω*_s_, *ω*_i_) in a Gaussian form, which is actually the case here. In this case, the eigen functions and eigen-value distribution of Eq. (2) are the Hermite polynomials and the thermal distribution, respectively, as shown in Appendix in the ref. 17. Then *g*^(2)^ and *g*^(3)^ can be expressed as follows^18^ (also see Methods), 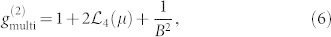

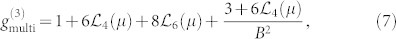
with 
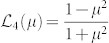
 and 
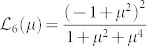
, where *B* is an optical gain coefficient defined by *r_k_* = *Bλ_k_* with the normalized mode weight 
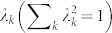
. The single parameter *μ* determines the thermal mode distribution as 
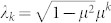
. The slope of 
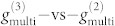
 can thus be defined as 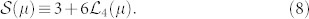
The red crosses in Fig. 4b show the *g*^(3)^ values as a function of *g*^(2)^. By fitting the slope of *g*^(3)^-vs-*g*^(2)^ to 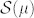 using the linear least squares fitting technique, we derived 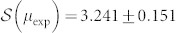. This corresponds to *μ*_exp_ = 0.961 ± 0.024. Once *μ*_exp_ is determined, each *B*_exp_ is obtained by eqs. (6) and (7) using corresponding *g*^(2)^ and *g*^(3)^, yielding 0.504 (for ~ 10 pJ) and 0.157 (for ~1 pJ). Note that *B*_exp_ can be independently determined by the measured average photon-number via the relation 
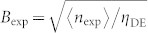
 which implies 0.506 (for ~ 10 pJ) and 0.148 (for ~ 1 pJ) agreeing well with the above results. Thus we can finally obtain the mode distribution of the squeezing parameter *r_k_* ( = *B*_exp_*λ_k_*). The amount of squeezing for each *r_k_* can be calculated via −10 log_10_(exp(−2*r_k_*)) [dB]. Reconstructed squeezing-level distributions (*k* = 0, 1, …, 40) are shown in Fig. 4c for a pump energy of ~10 pJ. The effective mode-number *K* of the squeezed vacua contained in the reconstructed distribution can be calculated using the relation 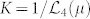, yielding 
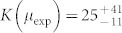
.

It is worth to compare *μ*_exp_ with its theoretical predictions. From the JSD characterized by the phase-matching condition of our nonlinear crystal and an envelope of the pump pulse, we can calculate the theoretical *μ* as *μ*_JSD_ ~ 0.99844 (see Methods for detailed calcuation) which is slightly deviated from *μ*_exp_. We also estimated the origin of the uncertainty of *μ*_exp_ by the Monte Carlo simulations with 1 × 10^7^ trials (same as that of the experiment. See Methods for details of the simulation). Figure 4d compares the experimental and simulated *g*^(3)^-vs-*g*^(2)^ plots, showing that those agree very well. Then from the simulated result, we obtained 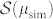 by linear least squares fitting which yields 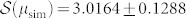 and *μ*_sim_ = 0.9973 ± 0.0212. We conclude that the uncertainty of our experimental data is mainly due to the statistical errors. For further investigation of the discrepancy between *μ*_exp_ and *μ*_JSD_, the setup should be further stabilized to allow a longer data acquisition for reducing the statistical errors.

## Discussion

In this work the nonclassical photon-number distributions as the even-odd photon-number oscillations were observed in an extremely broadband of 110 nm FWHM. This bandwidth is, to our knowledge, the broadest one whose nonclassicality was directly detected. The bands are technologically important ones, namely the telecom S-, C-, and L-bands. The mode reconstruction technique theoretically developed in the ref. 18 was applied, which inferred that the several tens of orthonormal modes were contained in every single optical pulse.

There are interesting open issues along with this work. First, the method used here is just for evaluating the mode weight distributions under certain assumptions on the SPDC spectral correlation. More sophisticated methods on mode analysis using direct detection with PNRDs are desired to be developed, as can be implemented by using homodyne detection^21^,^22^. Second, low-loss mode-separating devices should also be developed, which may include passive low-loss bandpass filters or recent proposals of quantum frequency conversion^42^,^43^, and combined with highly efficient PNRDs to detect and analyse mode characteristics of nonclassical states. Third, when a shaped broadband LO pulse^44^ is introduced in front of a PNRD, possibly combined with mode-separating devices, one can implement a displacement operation before a PNRD which could realize a phase-sensitive PNRD. A displaced squeezed state is known to show unique phase-dependent oscillation in photon-number distributions^45^,^46^, which has never been demonstrated yet. It also provides a means to implement quantum receivers to overcome the conventional limits of optical communications^4^,^5^,^6^,^47^.

Finally broadband light sources play essential roles in diverse fields in the classical domain, such as high-capacity optical communications^48^, optical coherence tomography^49^, optical spectroscopy^50^, and spectrograph calibration^51^. Supercontinuum sources in the vicinity of the 1550 nm telecommunication wavelength^52^ are of particular importance for wavelength division multiplexing in fibre-optic communications^53^. Our ultrabroadband source and detection scheme shown here could also provide a way to utilize the multimode nonclassical states for realizing such capabilities in the quantum domain.

## Methods

### Calibration of TES's detection efficiency depending on the wavelength

We used a continuous-wave (CW), wavelength-tunable ECDL (TSL-510, Santec) in order to measure the DE's wavelength dependence. The CW light beam from the ECDL was connected to the fibre-coupler. One of the outputs from the coupler was guided to our TES after passing through the variable attenuator. This power to the TES was heavily attenuated (~90 dB) such that the average power was set to be below 1 fW, and was monitored via the other output port of the coupler using a power meter, whose wavelength sensitivity was precisely calibrated. The coupler's splitting ratio and the degree of the attenuation were also precisely measured using the same power meter. Because photon arrival was random for the CW source, total counts from the TES was collected by the threshold detector (SR400, Stanford Research Systems). Photon counts from the TES were a few kHz, and the raw waveforms were not overlapped each other. We scanned the wavelength at each 3 nm step from 1512 to 1620 nm, and obtained the wavelength dependence of our TES's DE as shown in Fig. 2b.

### The second- and third-order correlation functions for the multimode squeezer

In this multimode “single-beam squeezer” case, the second- and third-order correlation functions are described in the ref. 18 as follows, 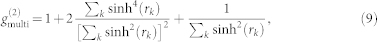

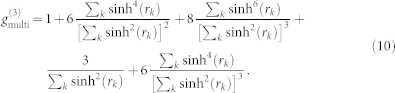


When a JSD takes a 2D Gaussian shape, *λ_k_* forms a thermal distribution defined by a single parameter *μ*, as 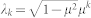. When the *r_k_* is small enough (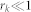), that is valid in our experimental conditions, the following approximations are valid as 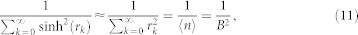

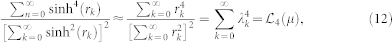

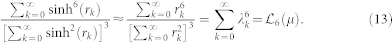
Then, eqs. (9) and (10) can be simplified into the eqs. (6) and (7). 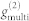 and 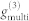 can be exactly calculated from the experimental photon statistics. Furthermore, from eqs. (6) and (7), 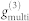 can be expressed as a function of 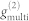 as 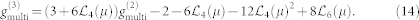
The slope of 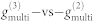 is finally defined as eq. (8).

### Specification of PPKTP crystal and SPDC spectrum

The JSD can be a product of two quantities as 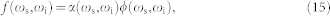
where *α*(*ω*_s_, *ω*_i_) describes pump-frequency envelop, and *ϕ*(*ω*_s_, *ω*_i_) describes phase-matching function for the SPDC in a nonlinear crystal^54^. Explicitly *α*(*ω*_s_, *ω*_i_) = sinc(*L*Δ*k*/2) with the crystal length *L* and phase mismatch Δ*k* = *k*_p_ − *k*_s_ − *k*_i_ − 2*π*/Λ. Λ denotes a grating period of the PPKTP crystals and 24.2 *μ*m in our case. Assuming that the transversal phase-matching is perfect, the longitudinal one can be described as Δ*k* = *k*_p,*z*_ − *k*_s,*z*_ − *k*_i,*z*_ − 2*π*/Λ, because we used collinear configuration where pump, signal, and idler are all in the same polarization. Note that the beam propagating direction is taken to be along *z*-axis and *k_μ_*(*ω_μ_*) = *ω_μ_n_z_*(*ω_μ_*)/*c* is the wave vectors. The frequencies are in a relation of *ω*_i_ = *ω*_p_ − *ω*_s_ = 2*ω*_0_ − *ω*_s_. The corresponding Sellmeier equation for *n_z_* can be found in the ref. 55. The blue line in Fig. 2c was theoretically derived using the above formulas and parameters.

The theoretical *K*_JSD_ can be calculated from the JSD using the singular value decomposition (SVD) technique^29^. The JSD was obtained from the above parameters and the pump linewidth of ~ 0.05 nm, and then discretized into a 16000 × 16000 square matrix with a frequency resolution of 2 GHz and with a frequency range of 179.3 THz (~ 1672 nm) to 211.3 THz (~ 1419 nm). We carried out SVD for the JSD matrix using MATLAB, and obtained *K*_JSD_ ~ 640. The corresponding *μ* is *μ*_JSD_ ~ 0.99844.

### Theoretical photon probability of multimode squeezed states for Monte Carlo simulations

In order to analyse the discrepancy between the experimentally obtained *μ*_exp_ and the theoretically obtained *μ*_JSD_, we carried out Monte Carlo simulations to illustrate how finite data size affects *S*(*μ*)-fitting errors. Before the simulations, we first calculate theoretical photon probabilities for the multimode squeezed vacua. The density matrix of the multimode vacuum state should be expressed as a tensor product of the decomposed squeezed vacuum states, whose squeezing parameters are characterized by *r_k_*( = *B*_exp_*λ_k_*). From this density matrix, one can derive the *n*-photon generation probabilities of the source. Though *n* is distributed to infinity, we truncated it at six photons which is a reasonable approximation to simulate our experimental situation in Fig 3b. The *n*-photon generation probabilities up to *n* = 6 are given by 
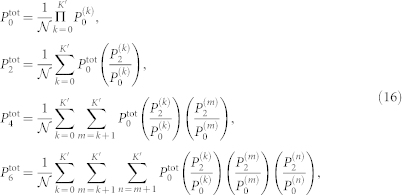
where 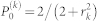, 
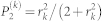
, and 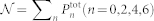. Here we assume that the *r_k_* is small enough and each mode only emits vacuum or two-photons. This assumption is valid when the mode number *K*′ is large enough. For example, in the case of four-photon emission, the number of cases for two-photon generations from the two different modes (*_K_*_′_*C*_2_ ~ *O*(*K*′^2^)) are much greater than those for four-photon emission from only one mode (*_K_*_′_*C*_1_ ~ *O*(*K*′)). Then the Monte Carlo simulation was performed along with eq. (16).

## Author Contributions

K.W. designed the whole setup, performed the experiment, and analysed data. Y.E. designed and built the OPA setup. H.B. designed and built the fast and automated data-acquisition system. T.Y. and S.I. characterized the OPA setup and provided assistance. D.F. and T.N. fabricated the TES device. K.W. and M.T. performed the theoretical analysis. K.E., D.F. and M.S. supervised the experiment. K.W., M.T. and M.S. wrote the manuscript with inputs from all the authors.
